# Assessment of Insulin Resistance in Subjects with Normal Glucose Tolerance, Hyperinsulinemia with Normal Blood Glucose Tolerance, Impaired Glucose Tolerance, and Newly Diagnosed Type 2 Diabetes (Prediabetes Insulin Resistance Research)

**DOI:** 10.1155/2016/9270768

**Published:** 2015-12-07

**Authors:** Guang Yang, Chunlin Li, Yanping Gong, Fusheng Fang, Hui Tian, Jian Li, Xiaoling Cheng

**Affiliations:** Department of Geriatric Endocrinology, Chinese PLA General Hospital, No. 28 Fuxing Road, Beijing 100853, China

## Abstract

*Aim.* To evaluate the differences in insulin resistance (IR) among subjects with normal glucose tolerance (NGT), hyperinsulinemia with NGT (HINS), impaired glucose tolerance (IGT), and newly diagnosed type 2 diabetes mellitus (T2DM).* Methods.* 5 NGT, 25 HINS, 25 IGT, and 25 T2DM subjects participated in this research. The hyperinsulinemic-euglycemic clamp technique (HECT) was performed in all of them to evaluate IR levels. The relative factors influencing IR were evaluated. The simple insulin sensitivity indices were calculated, and the correlation between each index and the M value was analyzed.* Results.* The *M* values of NGT, HINS, IGT, and T2DM groups were 11.88 ± 2.93 mg·kg^−1^·min^−1^, 6.23 ± 1.73 mg·kg^−1^·min^−1^, 6.37 ± 2.12 mg·kg^−1^·min^−1^, and 6.19 ± 1.89 mg·kg^−1^·min^−1^, respectively. *M* values in HINS, IGT, and T2DM groups were lower than those in the NGT group (*P* = 0.005); however, the differences among the HINS, IGT, and T2DM groups were not statistically significant (*P* = 0.835). The independent factors influencing the *M* value were waistline and fasting insulin level (FINS). The simple insulin sensitivity indices, especially Matsuda and Gutt index, were significantly associated with the *M* value (*P* < 0.01).* Conclusion.* IR existed in the HINS, IGT, and T2DM groups, and IR levels were consistent in the three groups. The independent factors influencing IR were waistline and FINS.

## 1. Introduction

Insulin resistance (IR) and impaired insulin secretion are the main pathogeneses in impaired glucose tolerance (IGT) and type 2 diabetes mellitus (T2DM). IR widely exists in many pathophysiological states. Several researchers have suggested that IR already exists before blood glucose abnormalities in diabetic patients [1, 2] and that hyperinsulinemia occurs before IGT shows several pathophysiological abnormalities. Therefore, several scholars have suggested that the T2DM process should be divided into the following three phases: hyperinsulinemia stage, prediabetes stage (IGT, IFG), and diabetes stage [1]. In other words, hyperinsulinemia and IGT are both reserve forces of T2DM. Hyperinsulinemia and IR are harmful even in subjects with NGT. For example, several researchers have indicated that a fasting plasma insulin level (FINS) of 39 mU/mL or greater was associated with a 31% increased risk of cardiovascular events in individuals without diabetes [3]. In the transition from normal to impaired and diabetic glucose tolerance, IR is the initiating agent. When the pancreatic beta cells produce enough insulin for compensation, blood glucose is maintained in the normal range; however, when the beta cells do not produce enough insulin to compensate for IR, the blood glucose level is inevitably elevated. Thus, hyperinsulinemia and IR exist long before IGT or T2DM occurs, and if physicians can provide an intervention treatment during the IGT period or in earlier stages such as the hyperinsulinemia stage, patients may have a better opportunity to prevent or delay the occurrence or development of diabetes and its complications.

In the present study, we evaluated the differences in IR levels among subjects with NGT, hyperinsulinemia with normal blood glucose tolerance (HINS), IGT, and newly diagnosed T2DM and analyzed the relatively dangerous factors of insulin sensitivity. The hyperinsulinemic-euglycemic clamp technique (HECT), which is considered the gold standard and is very expensive and complicated, was used to evaluate IR level. We also evaluated the relationship between the *M* value and other simple insulin sensitivity indices.

## 2. Materials and Methods

### 2.1. Study Subjects

Eighty adults participated in this study. Subjects were enrolled at the Chinese PLA General Hospital and were divided into four groups: NGT group (*n* = 5), HINS group (*n* = 25), IGT group (*n* = 25), and T2DM group (*n* = 25). The study was approved by the Medical Ethics Committee of the Chinese PLA General Hospital, and written informed consent was obtained from each subject before any study procedure was performed. This trial has been registered at chictr.org.cn (ChiCTR-ONRC-11001647).

### 2.2. Inclusion Criteria

*NGT group*: subjects were between 23 and 70 years of age; had a body mass index (BMI) of 19–23 kg·m^−2^; and had no history of heart, brain, or lung disease, high blood pressure, blood lipid disorders, chronic hepatitis, or liver cirrhosis, or family history of diabetes and hypertension; concentrations of blood fat, liver and kidney function, or blood uric acid were all within the normal range.* HINS*,* IGT*,* and T2DM groups*: subjects were between 23 and 70 years of age; had a BMI equal to or greater than 19 kg·m^−2^; and had no history of serious heart, brain, or lung disease, chronic hepatitis, or liver cirrhosis; liver function was not greater than 100 U/L; serum creatinine was <133 *μ*mol/L; and they had no endocrine or other diseases that influence glycometabolism. If the patient had a history of hypertension, blood pressure after treatment was controlled to a systolic blood pressure (SBP) <160 mmHg and diastolic blood pressure (DBP) <100 mmHg before being admitted.

#### 2.2.1. Diagnostic Criteria

Subjects were admitted to the Clinical Research Unit at 7:00 AM after fasting overnight. An oral glucose tolerance test (OGTT) was administered to all of the subjects. Blood samples were obtained to determine plasma glucose and insulin concentrations before (0 min) and after (120 min) consuming a 75 g glucose drink. Diagnostic criteria of glucose tolerance followed ADA and World Health Organization standards [4], and the diagnostic criteria of hyperinsulinemia were based on an epidemiological survey of our previous study in China [5, 6]. The detailed diagnostic criteria for the four groups are shown in Table 1. Subjects had not received any oral glucose-lowering drugs or insulin treatment. Diagnostic and clarification criteria of obesity followed the Chinese standard [7]: nonobese (BMI < 24 kg/m^2^), overweight (BMI ≥ 24 kg/m^2^, <28 kg/m^2^), and obese (BMI ≥ 28 kg/m^2^) and central obesity: waistline ≥90 cm in males, waistline ≥85 cm in females.

#### 2.2.2. Exclusion Criteria

Subjects should avoid administration of other medications known to affect insulin sensitivity within the past 6 months, including weight-loss drugs, Chinese herbal medicines, oral glucose-lowering drugs, and insulin. Patients with severe organ dysfunction or mental illness and type 1 diabetes mellitus also should be excluded.

### 2.3. Experimental Protocol

In the second visit after OGTT, subjects' height (cm), weight (kg), waist and hip circumference (cm), and blood pressure were measured, BMI (kg·m^−2^) and Waist-to-Hip Ratio (WHR) were calculated (BMI = weight × height^−2^, WHR = waistline/hip circumference), and blood samples were obtained at 7:00 AM after subjects fasted overnight to determine triglyceride (TG), total cholesterol (TC), high-density lipoprotein cholesterol (HDL-C), and low-density lipoprotein cholesterol (LDL-C). Urine specimens also were collected for urine microalbumin test. Blood pressure was measured using desktop mercury sphygmomanometer (purchase from Xinyuan Chang Medical Devices Co., Ltd., China). The waist circumference should be measured at the midpoint between the lower margin of the last palpable rib and the top of the iliac crest, using a stretch-resistant tape. Hip circumference should be measured around the widest portion of the buttocks, with the tape parallel to the floor (this method is suggested by WHO). Subjects' renal and liver functions and blood lipids concentrations were evaluated by the biochemistry department of our hospital (using Hitachi 7500 automatic biochemical analyzer, Japan). HbA1c was measured using Variant II HbA1c analyzer (Bio-Rad Laboratories, Hercules, CA, USA). HECT was performed in all of the subjects and the *M* value was calculated to estimate the IR levels. The differences in IR levels among the four groups were compared and the relative factors influencing the *M* value analyzed. The simple insulin sensitivity indices were calculated, and association of the simple indices with the gold standard of the IR *M* value was analyzed.

### 2.4. HECT Procedure

The study participants consumed a weight-maintaining diet containing at least 200 g carbohydrate per day for 3 days prior to the study. All of the studies were performed at 8:00 AM after a 12 h overnight fast. A catheter was inserted into an antecubital vein of one arm for intravenous infusion (infusion of insulin and glucose), and another catheter was inserted into a vein in the opposite arm, which was heated to 60°C using a thermostatically controlled box to obtain arterialized blood samples.* Insulin infusion*: the purpose of the euglycemic insulin clamp is to raise the plasma insulin concentration acutely to 100 *μ*U/mL above basal levels and to maintain it at this level for 120 min. At the beginning of the study, a basal blood sample was obtained, and the blood glucose concentration was tested. Next, the clamp system software calculated the total insulin dose needed during the entire process of the study according to the subject's body weight, basal blood glucose, and duration of the study, and the insulin was diluted with physiological saline into 50 mL solution. During the first 10 min, insulin was infused at a priming rate of 4 mL·kg^−1^·min^−1^, followed by infusion at a rate of 2 mL·kg^−1^·min^−1^ to the end of the study (120 min).* Glucose infusion*: glucose (20%) was infused at 10–120 min of the clamp procedure, and the infusion rate was adjusted every 5 min according to the subject's blood glucose concentration to maintain a plasma glucose concentration of 5 mmol/L. Blood samples were obtained every 10 min during the final 30 min of the clamp procedure to determine insulin and C-peptide concentrations. All of the blood samples were centrifuged; blood serum was separated and stored at −80°C and tested at the same time.* Sample processing and analyses*: HECT equipment consisting of system software, glucose analyzer, insulin infusion pump, and glucose infusion pump was purchased from EKF Diagnostics (Barleben, Germany). Plasma glucose was measured with an automated glucose analyzer using the glucose oxidase electrode method (BIOSEN5030 EKF-diagnostic GmbH, Magdeburg, Germany). Plasma insulin and C-peptide concentrations were measured by chemiluminescence assay (IMMULITE1000, Siemens Healthcare, Erlangen, Germany).* Calculation*: insulin sensitivity is evaluated by “*M*” value; the *M* value was calculated according to the average exogenous glucose infusion rate divided by weight in kilograms during the last 30 minutes of the 120-minute clamp [8, 9].

### 2.5. Calculations according to OGTT

Simple insulin sensitivity indices and islet beta cell function were calculated through the follow equations, shown in Table 2.

### 2.6. Statistical Analyses

All of the measured parameters were determined as means ± standard deviation (SD) values. The normality of the data was tested using SPSS version 10.0 software (SPSS, Inc., Chicago, IL, USA). Differences among the four groups were compared using analysis of variance (ANOVA) (for normally distributed variables) or the Kruskal-Wallis *H* test (for nonnormal distribution of variables). The independent samples *t*-test was used to compare each parameter between any two patient groups. Linear correlation and stepwise regression analyses were used to assess the relationship between variables. A *P* value <0.05 was considered statistically significant. The number of subjects is indicated by *n*. Statistical analyses were performed using SPSS version 10.0 software.

## 3. Results

### 3.1. Subjects' Baseline Data

Study subjects' characteristics and metabolic variables, including age, gender, BMI, waistline, WHR, blood lipids, blood pressure, plasma insulin, and blood glucose levels, are shown in Table 3.

### 3.2. Evaluation of IR Levels in the Four Groups

The HECT was successfully performed in the four groups (see [9]). The IR level was represented by the *M* value (calculated by the average glucose infusion rate during the last 30 min of the clamp procedure). The *M* values in the NGT, HINS, IGT, and T2DM groups were 11.88 ± 2.93 mg·kg^−1^·min^−1^, 6.23 ± 1.73 mg·kg^−1^·min^−1^, 6.37 ± 2.12 mg·kg^−1^·min^−1^, and 6.19 ± 1.89 mg·kg^−1^·min^−1^, respectively. ANOVA results showed that *M* values in the HINS, IGT, and T2DM groups were lower than those in the NGT group, and the differences were statistically significant (*P* = 0.005); however, the differences among the HINS, IGT, and T2DM groups were not statistically significant (*P* = 0.835; Table 3).

### 3.3. Risk Factors Analyzing of *M* Value

#### 3.3.1. The Related Influencing Factors of *M* Value

Using *M* value as the dependent variable and potential influencing factors as independent variables, based on the linear correlation analysis results, showed that the *M* values were linearly correlated with waistline, FINS, BMI, body weight, HDL, TG, insulin level at 120 min in the OGTT (Ins 120), and age. *M* values were not associated with the WHR, glucose level at 120 min in the OGTT (Glu 120), Glu 0, urine microalbumin (ALB), HbA1c, LDL, TC, SBP, or DBP. The correlation coefficients and *P* values are shown in Table 4.

#### 3.3.2. Independent Factors Influencing the *M* Value

*M* values can be affected by many of the influencing factors mentioned above. Using a stepwise regression equation, the absolute value of the correlation coefficient above 0.4 was entered, and the mutual influence between variables was removed. The equation results showed waistline and FINS with a model correlation coefficient of 0.58 (*P* < 0.01) and regression coefficients of the independent variables of −0.39 and −0.30 (Table 5).

### 3.4. Subgroup Analysis

#### 3.4.1. Subgroup Analyses according to BMI

Subjects in the HINS, IGT, and T2DM groups were divided into three subgroups according to BMI levels (nonobesity, overweight, and obesity subgroups). Results showed that the *M* values decreased significantly with increased BMI (*P* values were 0.000, 0.025, 0.0026, resp., in the HINS, IGT, and T2DM groups), but at the same BMI levels, the *M* values were not significantly different among the three groups. We also calculated the Matsuda index in each group and get the similar conclusion with *M* value (Table 6).

#### 3.4.2. Subgroup Analyses according to Waistline

Subjects in the HINS, IGT, and T2DM groups were divided into two subgroups according to waist circumference (non-central-obesity subgroup and central-obesity subgroup). Results showed the *M* values decreased significantly with increased waistline (*P* < 0.05), but when the waistlines were similar, the *M* values were not significantly different among the three groups. We also calculated the Matsuda index in each group and get the similar conclusion with *M* value (Table 7).

#### 3.4.3. Subgroup Analyses according to Fasting Hyperinsulinemia

In HINS, IGT, and T2DM groups, the *M* values in the fasting hyperinsulinemia subgroup (fasting insulin level >15 *μ*IU/mL) were lower than those in the nonfasting hyperinsulinemia subgroup (fasting insulin level <15 *μ*IU/mL). The differences between the subgroups were not statistically significant in the HINS group (*P* = 0.35) but were significant in the IGT and T2DM groups (*P* = 0.00 and *P* = 0.03, resp.), while, at the same fasting insulin levels, the *M* values were not significantly different among the three groups (*P* = 0.55). We also calculated the Matsuda index in each group and get the similar conclusion with *M* value (Table 8).

### 3.5. Assessment of Other Simple Insulin Sensitivity Indices

The simple insulin sensitivity indices Matsuda index, Gutt index, HOMA-IR, QUICKI, FINS, and FINS/FPG were significantly associated with the *M* value and the correlation coefficients (*r*) were as follows: 0.56, 0.54, −0.51, 0.48, −0.46, and −0.35, respectively (*P* < 0.01; Table 9).

### 3.6. Evaluation of Islet Beta Cell Function

The islet cell function indices of the four groups were calculated using HOMA-*β*, disposition index, and insulinogenic index. Results showed that when comparing HINS, IGT, and T2DM groups, HOMA-*β*, disposition index, and insulinogenic index values in the HINS group were all higher than those in the IGT and T2DM groups (*P* < 0.01). The above indexes in the IGT group were also higher than those in the T2DM group (*P* < 0.01).

## 4. Discussion

IR exists across many pathological conditions, such as obesity, hypertension, lipid metabolic disorder, polycystic ovary syndrome, and acanthosis nigricans. IR is one of the most important factors in the process of occurrence and development of T2DM. Studies have shown that IR exists even in physiological conditions. For example, glucose clamp technique was performed by Reaven [19] on 100 nonobese individuals with normal oral glucose tolerance. Subjects were divided into four groups according to the *M* value. Results showed that the group with low *M* values represented approximately one-third of the high *M* value group, indicating that IR is present in approximately 25% of nonobese normal oral glucose tolerance individuals. Thus, some scholars suggested that hyperinsulinemia and IR exist prior to the occurrence of blood glucose abnormalities [1, 2]. During the course of the disease, IR is the initiating factor in the majority of patients with T2DM. In these conditions, deterioration of glucose tolerance can only be prevented if beta cells increase their insulin secretory response and maintain a state of chronic hyperinsulinemia. When this is not achieved, an increase of blood glucose inevitably occurs [1, 2]. Therefore, Groop [1] suggested that the process of T2DM be divided into three phases: hyperinsulinemia stage, prediabetes stage (IGT, IFG), and diabetes stage. We investigated what changes occurred in IR levels and the differences among groups during the T2DM transition process. The HECT was used to measure insulin sensitivity in the four groups, and our results showed that the *M* values in NGT, HINS, IGT, and T2DM groups were 11.88 ± 2.93 mg·kg^−1^·min^−1^, 6.23 ± 1.73 mg·kg^−1^·min^−1^, 6.37 ± 2.12 mg·kg^−1^·min^−1^, and 6.19 ± 1.89 mg·kg^−1^·min^−1^, respectively. Among them, the *M* values in the HINS, IGT, and T2DM groups were lower than those in the NGT group but were not different among the HINS, IGT, and T2DM groups, indicating that IR already existed in the HINS group. Although glucose tolerance levels were different in the HINS, IGT, and T2DM groups, the IR was the same in the three groups. Our results are in agreement with numerous comparison studies. For example, Chen et al. [20] evaluated insulin sensitivity in obese adults with NGT and T2DM adults using HECT. The results showed that the *M* values were not different between those subjects. Another study also showed that IR existed and was similar in both the IFG and T2DM groups [21]. However, Bacha et al. [22] observed that insulin sensitivity was lower in individuals with IFG or/and IGT and was further decreased in subjects with T2DM when compared to obese adolescents with NGT. In the present study, hyperinsulinemia individuals with NGT were classified as a separate group. Our results showed that the IR levels in the HINS group were similar to those in the IGT and T2DM groups; the experimental design was different from previously mentioned studies and has not been reported elsewhere. We emphasized hyperinsulinemia because in our previous studies subjects with HINS were followed up for 2 years, and 18.6% of patients converted from NGT to IGT or IFG, and 2.3% developed DM. The incidence was much higher than that in nonhyperinsulinemia subjects with NGT (5.4% and 0.7%, *P* < 0.01), illustrating that the probability of conversion from NGT to DM significantly increased when HINS was diagnosed [4]. Another study showed similar results; 515 healthy normoglycemic adults with hyperinsulinemia were followed up for 24 years. Half of the participants developed dysglycemia by the end of the study. Analysis showed that the most significant predictor of progression to dysglycemia was hyperinsulinemia [23, 24]. Hyperinsulinemia is harmful in subjects with normal or abnormal glucose tolerance. The Helsinki Policemen Study [25], the Busselton Study [26], the Wisconsin Epidemiologic Study [27], and the RISC study [28] showed that high plasma insulin, fasting or after oral glucose load, was associated with increased risk of major CHD events independently of other conventional cardiovascular risk factors (including blood glucose, cholesterol, triglycerides, blood pressure, indices of obesity, smoking, and physical activity). Kelly et al. [29] suggested that increased serum insulin levels be used as a clinical marker in a primary care setting for early diagnosis and preventative care, which may be beneficial for patients at high risk of diabetes.

Therefore, based on our study results and the abovementioned literature review, consistent results regarding IR levels among NGT, IGT, and T2DM groups were not obtained, and differences among groups existed. Differences in baseline characteristics of study populations are the most likely reason for the conflicting results in several studies. Characteristics considered included age, obesity, and blood fat. Next, we analyzed the factors influencing IR. The results showed that *M* value was associated with multiple metabolic indices. Based on stepwise regression analysis, the independent factors influencing the *M* value were waistline and FINS, which is consistent with other literature reports [30, 31]. Subgroup analysis was further performed according to the related risk factors. Results showed that, with the same level of glucose tolerance, the *M* value tended to gradually decline when BMI and waistline increased, indicating that obesity, especially central obesity, plays an important role in IR. Results from the subgroup analysis according to fasting insulin level also showed that *M* values in individuals with fasting hyperinsulinemia were lower than those without fasting hyperinsulinemia, confirming that waist circumference and FINS were predictors of IR levels.

In the present study, there was no systematic evaluation of islet beta cell function in the three groups, only rough estimates of the beta cell function using HOMA-*β*, disposition index, and insulinogenic index. Results showed that, with glucose tolerance progression (from HINS to IGT then to T2DM), beta cell function gradually deteriorates. These results confirmed the theory of Reaven [1, 2]. For the HINS population, due to the good compensatory function of beta cells, these subjects did not develop glucose metabolic disorders. HOMA-*β* is calculated using FINS; thus, beta cell function is often overestimated due to the interference of IR. Therefore, in our data, HOMA-*β* in HINS and IGT groups were higher than in the NGT group. However, among HINS, IGT, and T2DM groups, because they had the same IR level according to our previous data, the interference of IR was negligible and HOMA-*β* could effectively evaluate the islet cell function difference between the three groups. The disposition index could remove the interference of IR and could get a more accurate evaluation on beta cell function.

The HECT used to assess insulin sensitivity is time consuming, expensive, and stressful for the participants and is generally only used for small samples. In clinical practice, all types of simple insulin sensitive indices are commonly used to evaluate IR, which correlate well with the glucose clamp technique in many studies [12, 14, 32]. We used the simple insulin sensitive index to evaluate the IR levels in the four groups and obtained the same results as in the *M* value evaluation. Based on regression analysis, the correlation coefficients of the absolute value of each index and *M* value were between 0.35 and 0.56. Among them, Matsuda index and Gutt index correlated best with the *M* value (*r* = 0.56 and 0.54, *P* < 0.01). However, these indices are limited by the narrow scope of information, depend on the accuracy of insulin determination method, are not applicable for individuals with severely damaged beta cells, and are more suitable for epidemiological investigations of IR using large cohorts.

Of course, there are also some disadvantages in our study, the number of normal subjects was only 5; we feel regret about this, for although the inclusion criteria of age were between 23 and 70 years old most of the subjects in HINS, IGT, and T2DM groups were middle aged and elderly people; in order to match the age, only a very few volunteers finally meet the NGT inclusion criteria; thus the enrollment of NGT group is really hard for us; that is the reason why the number of the subjects in NGT is very few.

## 5. Conclusions

IR existed in HINS, IGT, and T2DM groups and was the same in the three groups. The independent factors influencing IR were waistline and FINS. Several simple insulin sensitivity indices especially Matsuda index and Gutt index were significantly associated with the *M* value and were applicable to evaluate IR. The beta cell function decreased in procedure from HINS to IGT then to T2DM.

## Figures and Tables

**Table 1:** Diagnostic criteria.

	NGT	HINS	IGT	T2DM
Glu 0 (mmol/L)	<6.1	<6.1	≥6.1–<7.0	≥7.0
Glu 120 (mmol/L)	<7.8	<7.8	≥7.8–<11.1	≥11.1
Ins 0 (mU/L)	<15	≥15	—	—
Ins 120 (mU/L)	<80	≥80	—	—
HbA1c (%)	—	—	—	<10

**Table 2:** Indices of insulin sensitivity and *β* cell function derived from OGTT measurements of glucose and insulin.

Index	Formula	Reference
HOMA-IR	[FPG (mmol/L) × FINS (mU/L)]/22.5	[10, 11]
Insulin/glucose ratio	FINS (mU/L)/FPG (mmol/L)	[12, 13]
QUICKI	1/[LogFINS (mU/L) + LogFPG (mg/dL)]	[14]
Matsuda index	10000/[FPG (mg/dL) × FINS (mU/L) × (mean glucose × mean insulin)]^1/2^	[15]
Gutt index	{75000 + [GLU0 (mg/dL) − GLU120] × 0.19 × body weight (kg)}/{120 × log [mean insulin (mU/L)] × mean glucose}	[16]
HOMA-*β*	20 × FINS (mU/L)/[FPG (mmol/L) − 3.5]%	[10, 11]
Disposition index	HOMA-*β*/HOMA-IR	[17]
Insulinogenic index	[INS120 (mU/L) − INS0]/[GLU120 (mmol/L) − GLU0]	[18]

**Table 3:** Baseline data of the subjects—mean ± SE (*n*).

	NGT (*n* = 5)	HINS (*n* = 25)	IGT (*n* = 25)	T2DM (*n* = 25)	*P*
Age (years)	44.40 ± 11.74	43.36 ± 11.24	45.56 ± 9.83	44.40 ± 8.18	0.77
Gender (F/M)	1/4	4/21	4/21	5/20	0.99
BMI (kg/m^2^)	22.41 ± 1.54	27.26 ± 3.60^*∗∗*^	27.38 ± 3.57^*∗∗*^	26.49 ± 3.52^*∗∗*^	0.043
Waist (cm)	76.75 ± 7.72	89.71 ± 10.92^*∗∗*^	91.26 ± 9.85^*∗∗*^	91.68 ± 10.00^*∗∗*^	0.057
WHR	0.90 ± 0.11	0.94 ± 0.10	0.93 ± 0.06	0.95 ± 0.06	0.52
SBP (mmHg)	124.50 ± 11.82	124.92 ± 12.03	120.76 ± 12.15	126.32 ± 16.04	0.63
DBP (mmHg)	74.00 ± 7.30	79.88 ± 6.95	80.08 ± 9.24	78.36 ± 10.85	0.56
TG (mmol/L)	1.24 ± 0.73	2.40 ± 1.43^*∗*^	2.05 ± 1.16^*∗*^	1.77 ± 0.81	0.13
HDL (mmol/L)	1.62 ± 0.35	1.38 ± 0.32	1.21 ± 0.38^*∗∗*##^	1.06 ± 0.21^*∗∗*##^	0.000
LDL (mmol/L)	2.67 ± 0.67	2.68 ± 0.61	2.95 ± 0.89	3.14 ± 0.80	0.175
TC (mmol/L)	4.89 ± 0.54	5.44 ± 0.80	4.83 ± 0.87	4.97 ± 0.88	0.15
Glu 0 (mmol/L)	4.89 ± 0.59	4.95 ± 0.43	5.39 ± 0.60^*∗∗*##^	8.09 ± 1.67^*∗∗*##@@^	0.000
Glu 120 (mmol/L)	5.58 ± 1.40	6.22 ± 1.00	8.92 ± 1.06^*∗∗*##^	13.15 ± 3.62^*∗∗*##@@^	0.000
FINS (mU/L)	5.29 ± 4.61	11.69 ± 5.98^*∗∗*^	11.87 ± 6.80^*∗∗*^	8.79 ± 6.59^*∗∗*#@^	0.029
ALB/CR (mg/g)	10.50 ± 3.54	10.57 ± 6.86	20.69 ± 18.05	15.90 ± 20.68	0.756
HbA1c (%)	—	—	5.59 ± 0.35	8.02 ± 2.03^@@^	0.000
*M* value (mg·kg^−1^·min^−1^)	11.88 ± 2.93	6.23 ± 1.73^**∗∗**^	6.37 ± 2.12^**∗∗**^	6.19 ± 1.89^**∗∗**^	0.005

Notes: differences among the four groups were compared using analysis of variance (ANOVA) or the Kruskal-Wallis *H* test; the *P* values were shown in the right column. Then we compare each parameter between any two patient groups, ^*∗*^ *P* < 0.05, ^*∗∗*^ *P* < 0.01 compared with NGT, ^#^ *P* < 0.05, ^##^ *P* < 0.01 compared with HINS, and ^@^ *P* < 0.05, ^@@^ *P* < 0.01 compared with IGT.

**Table 4:** Linear correlation analysis.

Variables	Correlation coefficients	*P*
Waistline	−0.51	0.000
FINS	−0.46	0.000
BMI	−0.50	0.000
Body weight	−0.50	0.000
HDL	0.34	0.002
TG	−0.28	0.012
Ins 120	−0.37	0.012
Age	−0.14	0.019
WHR	—	0.099
Glu 120	—	0.077
Glu 0	—	0.48
HbA1c	—	0.39
LDL	—	0.75
TC	—	0.56
ALB	—	0.16
SBP	—	0.99
DBP	—	0.15

**Table 5:** Stepwise regression analyses.

Variables	Regression coefficients	Correlation coefficient	*P*
Waistline	−0.39	0.58	0.000
FINS	−0.30		

**Table 6:** Subgroup analyses according to BMI—mean ± SE (*n*).

	NGT (*n* = 5)	HINS (*n* = 25)			IGT (*n* = 25)			T2DM (*n* = 25)		
		Nonobesity (*n* = 5)	Overweight (*n* = 14)	Obesity (*n* = 6)	Nonobesity (*n* = 4)	Overweight (*n* = 12)	Obesity (*n* = 9)	Nonobesity (*n* = 7)	Overweight (*n* = 12)	Obesity (*n* = 6)
Weight (Kg)	62.20 ± 6.75	63.38 ± 4.23	76.04 ± 6.64^*∗∗*^	91.50 ± 12.80^*∗∗*##^	63.50 ± 3.63	72.63 ± 7.56^*∗∗*^	91.50 ± 9.57^*∗∗*##^	62.43 ± 8.94	71.38 ± 4.25^*∗∗*^	90.42 ± 7.872^*∗∗*##^
BMI (kg/m^2^)	22.41 ± 1.54	22.90 ± 0.65	26.39 ± 0.88^*∗∗*^	32.66 ± 1.56^*∗∗*##^	22.72 ± 0.58	25.98 ± 1.39^*∗∗*^	31.32 ± 2.05^*∗∗*##^	2.88 ± 0.81	26.05 ± 1.29^*∗∗*^	31.57 ± 2.29^*∗∗*##^
Waist (cm)	76.75 ± 7.72	77.75 ± 9.11	88.50 ± 6.76^*∗*^	102.25 ± 7.39^*∗∗*##^	77.88 ± 6.43	89.46 ± 5.53^*∗∗*^	99.61 ± 7.79^*∗∗*##^	82.29 ± 5.09	90.08 ± 4.40^*∗∗*^	105.83 ± 6.05^*∗∗*##^
TG (mmol/L)	1.24 ± 0.73	2.13 ± 1.12	2.67 ± 1.64	2.14 ± 1.30	1.33 ± 0.81	1.88 ± 1.31	2.59 ± 0.90^*∗*#^	1.31 ± 0.54	1.80 ± 0.81	2.26 ± 0.87^*∗∗*^
HDL (mmol/L)	1.62 ± 0.35	1.36 ± 0.19	1.41 ± 0.38	1.35 ± 0.29	1.34 ± 0.18	1.32 ± 0.48	1.04 ± 0.23^*∗*^	1.20 ± 0.16	1.06 ± 0.20	0.88 ± 0.15^*∗∗*#^
FINS (mU/L)	5.29 ± 4.61	6.25 ± 1.64	11.39 ± 5.99^*∗*^	15.45 ± 5.82^*∗∗*^	7.20 ± 4.94	10.16 ± 6.04^*∗*^	16.21 ± 6.56^*∗*#^	3.52 ± 0.88	9.91 ± 6.996^*∗∗*^	12.701 ± 6.31^*∗∗*^
*M* value	11.88 ± 2.93	7.74 ± 1.48	6.19 ± 1.19^*∗*^	5.86 ± 2.18^*∗*^	8.40 ± 1.38	6.59 ± 2.17	5.18 ± 1.59^*∗∗*#^	7.64 ± 1.29	6.44 ± 1.59^*∗*^	4.00 ± 0.77^*∗∗*##^
Matsuda index	13.16 ± 7.24	6.28 ± 0.49	4.88 ± 2.26^*∗*^	4.12 ± 1.50^*∗*^	10.92 ± 4.64	6.57 ± 2.97^*∗*^	4.11 ± 2.41^*∗∗*#^	10.20 ± 3.21	5.29 ± 2.57^*∗∗*^	3.54 ± 1.24^*∗∗*#^

Notes: comparison among HINS, IGT, and T2DM groups, ^*∗*^ *P* < 0.05, ^*∗∗*^ *P* < 0.01 compared with nonobesity subgroups and ^#^ *P* < 0.05, ^##^ *P* < 0.01 compared with overweight subgroups.

**Table 7:** Subgroup analyses according to waistline—mean ± SE (*n*).

	NGT (*n* = 5)		HINS (*n* = 25)	IGT (*n* = 25)		T2DM (*n* = 25)	
		Non-central-obesity (*n* = 11)	Central-obesity (*n* = 14)	Non-central-obesity (*n* = 10)	Central-obesity (*n* = 15)	Non-central-obesity (*n* = 10)	Central-obesity (*n* = 15)
Age	44.40 ± 11.74	42.82 ± 10.03	44.91 ± 13.72	48.20 ± 5.55	43.80 ± 11.72	44.90 ± 8.74	44.07 ± 8.08
Weight	62.20 ± 6.75	69.68 ± 6.54	83.14 ± 9.48^*∗∗*^	65.65 ± 5.40	86.17 ± 10.14^*∗∗*^	67.85 ± 7.42	77.17 ± 13.63^*∗*^
BMI	22.41 ± 1.54	24.94 ± 1.86	29.34 ± 3.02^*∗∗*^	24.79 ± 2.51	29.10 ± 3.14^**∗∗**^	23.98 ± 1.68	28.16 ± 3.45^*∗∗*^
Waistline	76.75 ± 7.72	81.36 ± 6.71	97.18 ± 5.66^*∗∗*^	82.00 ± 6.04	97.43 ± 6.38^*∗∗*^	84.60 ± 5.15	96.40 ± 9.74^*∗∗*^
WHR	0.90 ± 0.11	0.89 ± 0.09	0.99 ± 0.08^*∗*^	0.90 ± 0.06	0.96 ± 0.05^*∗*^	0.93 ± 0.06	0.98 ± 0.05^*∗*^
TG	1.24 ± 0.73	2.45 ± 1.64	2.52 ± 1.34	1.67 ± 0.99	2.30 ± 1.23	1.42 ± 0.41	2.01 ± 0.93^*∗*^
HDL	1.62 ± 0.35	1.42 ± 0.24	1.32 ± 0.39	1.35 ± 0.39	1.12 ± 0.37	1.13 ± 0.25	1.00 ± 0.17^##^
FINS	5.29 ± 4.61	9.34 ± 6.11	13.10 ± 5.49	7.84 ± 5.41	14.55 ± 6.42^*∗*^	7.46 ± 8.06	9.68 ± 5.54^@^
*M* value	11.88 ± 2.93	6.92 ± 1.43	5.53 ± 1.21^*∗*^	7.81 ± 1.25	5.24 ± 1.71^*∗∗*^	**7.26 ± 1.70**	5.48 ± 1.69^*∗*^
Matsuda index	13.16 ± 7.24	5.75 ± 2.35	4.12 ± 1.21^*∗*^	8.92 ± 3.85	4.69 ± 2.63^**∗∗**^	8.26 ± 4.29	4.90 ± 2.28^*∗*^

Notes: comparison among HINS, IGT, and T2DM groups, ^*∗*^ *P* < 0.05, ^*∗∗*^ *P* < 0.01 compared with non-central-obesity subgroups; in central-obesity subgroups, comparison among different glucose tolerance groups, ^##^ *P* < 0.01 compared with HINS-central-obesity group and ^@^ *P* < 0.05, compared with IGT-central-obesity group.

**Table 8:** Subgroup analyses according to fasting insulin level—mean ± SE (*n*).

	NGT (*n* = 5)	HINS (*n* = 25)		IGT (*n* = 25)		T2DM (*n* = 25)	
		FINS < 15 (*n* = 18)	FINS ≥ 15 (*n* = 7)	FINS < 15 (*n* = 16)	FINS ≥ 15 (*n* = 9)	FINS < 15 (*n* = 22)	FINS ≥ 15 (*n* = 3)
Weight	62.20 ± 6.75	75.50 ± 10.66	82.86 ± 14.32	72.13 ± 10.45	88.33 ± 11.60^*∗∗*^	84.67 ± 14.79	84.67 ± 14.79
BMI	22.41 ± 1.54	26.38 ± 2.80	29.39 ± 4.60^*∗*^	25.76 ± 2.76	30.26 ± 3.07^*∗∗*^	26.09 ± 3.43	29.41 ± 3.18
Waistline	76.75 ± 7.72	87.21 ± 9.90	95.79 ± 11.62	87.41 ± 8.78	98.11 ± 7.99^*∗∗*^	88.62 ± 10.90	93.17 ± 12.02
WHR	0.90 ± 0.11	0.93 ± 0.10	0.95 ± 0.12	0.92 ± 0.06	0.96 ± 0.06	0.95 ± 0.06	0.98 ± 0.08
TG	1.24 ± 0.73	2.80 ± 1.52	1.44 ± 0.35^*∗∗*^	1.49 ± 0.69	3.04 ± 1.19^*∗∗*##^	1.78 ± 0.86	1.70 ± 0.22^@^
HDL	1.62 ± 0.35	1.35 ± 0.35	1.47 ± 0.21	1.36 ± 0.39	0.98 ± 0.24^*∗∗*##^	1.07 ± 0.22	0.98 ± 0.02^##^
FINS	5.29 ± 4.61	8.78 ± 4.20	18.74 ± 2.86^*∗∗*^	7.82 ± 3.76	19.07 ± 4.60^*∗∗*^	6.80 ± 3.56	23.37 ± 5.12^*∗∗*^
*M* value	11.88 ± 2.93	6.57 ± 1.40	5.34 ± 2.28	7.53 ± 1.44	4.31 ± 1.43^*∗∗*^	6.43 ± 1.78	4.48 ± 2.11^*∗*^
Matsuda index	13.16 ± 7.24	5.36 ± 2.05	3.64 ± 0.95^*∗*^	8.28 ± 3.34	3.00 ± 0.94^*∗∗*^	6.87 ± 3.35	1.69 ± 0.49^*∗∗*^

Notes: comparison among HINS, IGT, and T2DM groups, ^*∗*^ *P* < 0.05, ^*∗∗*^ *P* < 0.01 compared with nonfasting hyperinsulinemia subgroup; in fasting hyperinsulinemia subgroup, comparison among different glucose tolerance groups, ^##^ *P* < 0.01 compared with HINS and ^@^ *P* < 0.05, compared with IGT.

**Table 9:** Assessment of islet beta cell function and other simple insulin sensitivity indexes—mean ± SE (*n*).

	NGT	HINS	IGT	T2DM	*P*
FINS	5.29 ± 4.61	11.69 ± 5.98^*∗∗*^	11.87 ± 6.80^*∗∗*^	8.79 ± 6.59^*∗∗*#@^	0.029
FINS/FBG	1.02 ± 0.75	2.37 ± 1.22^*∗*^	2.27 ± 1.34^*∗*^	1.15 ± 0.89^##@@^	0.001
HOMA-IR	0.99 ± 1.21	2.59 ± 1.38^*∗∗*^	2.78 ± 1.57^*∗∗*^	3.07 ± 2.29^*∗∗*^	0.006
QUICKI	0.40 ± 0.05	0.34 ± 0.04^*∗∗*^	0.34 ± 0.03^*∗∗*^	0.34 ± 0.03^*∗*^	0.016
Matsuda index	13.16 ± 7.24	4.86 ± 1.95^*∗∗*^	6.38 ± 3.75^*∗∗*^	6.24 ± 3.58^*∗∗*^	0.0005
Gutt index	5.10 ± 1.29	3.62 ± 0.59^*∗∗*^	3.48 ± 0.0.80^*∗∗*^	2.77 ± 0.95^*∗∗*##@@^	0.000
HOMA-*β*	70.32 ± 32.43	176.96 ± 115.28^*∗*^	143.89 ± 93.47^*∗*##^	44.11 ± 35.50^*∗*##@@^	0.000
Disposition index	77.34 ± 34.56	71.23 ± 29.44	50.40 ± 19.69^*∗∗*##^	14.76 ± 7.07^*∗∗*##@@^	0.000
Insulinogenic index	69.02 ± 75.94	99.26 ± 96.58	15.68 ± 18.04^*∗∗*##^	8.56 ± 7.20^*∗∗*##@@^	0.000
*M* value	11.88 ± 2.93	6.23 ± 1.73^*∗∗*^	6.37 ± 2.12^*∗∗*^	6.19 ± 1.89^*∗∗*^	0.005

Notes: differences among the four groups were compared using analysis of variance (ANOVA) or the Kruskal-Wallis *H* test; the *P* values were shown in the right column. Then we compare each parameter between any two patient groups, ^*∗*^ *P* < 0.05, ^*∗∗*^ *P* < 0.01 compared with NGT, ^#^ *P* < 0.05, ^##^ *P* < 0.01 compared with HINS, and ^@^ *P* < 0.05, ^@@^ *P* < 0.01 compared with IGT.
